# G-CSFR Ubiquitination Critically Regulates Myeloid Cell Survival and Proliferation

**DOI:** 10.1371/journal.pone.0003422

**Published:** 2008-10-16

**Authors:** Jing Ai, Lawrence J. Druhan, Megan J. Loveland, Belinda R. Avalos

**Affiliations:** 1 Heart and Lung Research Institute, The Ohio State University, Columbus, Ohio, United States of America; 2 Division of Hematology/Oncology, The Ohio State University, Columbus, Ohio, United States of America; Ordway Research Institute, United States of America

## Abstract

The granulocyte colony-stimulating factor receptor (G-CSFR) is a critical regulator of granulopoiesis. Mutations in the G-CSFR in patients with severe congenital neutropenia (SCN) transforming to acute myelogenous leukemia (AML) have been shown to induce hypersensitivity and enhanced growth responses to G-CSF. Recent studies have demonstrated the importance of the ubiquitin/proteasome system in the initiation of negative signaling by the G-CSFR. To further investigate the role of ubiquitination in regulating G-CSFR signaling, we generated a mutant form of the G-CSFR (K762R/G-CSFR) which abrogates the attachment of ubiquitin to the lysine residue at position 762 of the G-CSFR that is deleted in the Δ716 G-CSFR form isolated from patients with SCN/AML. In response to G-CSF, mono-/polyubiquitination of the G-CSFR was impaired in cells expressing the mutant K762R/G-CSFR compared to cells transfected with the WT G-CSFR. Cells stably transfected with the K762R/G-CSFR displayed a higher proliferation rate, increased sensitivity to G-CSF, and enhanced survival following cytokine depletion, similar to previously published data with the Δ716 G-CSFR mutant. Activation of the signaling molecules Stat5 and Akt were also increased in K762R/G-CSFR transfected cells in response to G-CSF, and their activation remained prolonged after G-CSF withdrawal. These results indicate that ubiquitination is required for regulation of G-CSFR-mediated proliferation and cell survival. Mutations that disrupt G-CSFR ubiquitination at lysine 762 induce aberrant receptor signaling and hyperproliferative responses to G-CSF, which may contribute to leukemic transformation.

## Introduction

The granulocyte colony-stimulating factor receptor (G-CSFR) plays a critical role in myeloid cell survival, proliferation, and neutrophilic maturation [1]–[8]. Like other members of the cytokine receptor family, the G-CSFR undergoes conformational changes in response to G-CSF binding, leading to activation of associated Jak kinases and downstream signaling cascades that include signal transducer and activators of transcription (Stat) kinases, Ras/mitogen-associated protein (Ras/MAP) kinase, and the phosphatidylinositol-3 (PI3) kinase/Akt pathways [9]–[10]. Simultaneously, mechanisms that down-regulate the G-CSFR in target cells are rapidly turned on to attenuate receptor signaling and protect cells from over-stimulation. Although multiple processes, including the recruitment of SHIP and SHP-1 to the G-CSFR complex as well as the production of SOCS proteins [11]–[13] have been extensively studied, the mechanisms mediating negative G-CSFR signaling remain poorly understood.

Ligand binding has been shown to trigger intracellular signaling as well as endocytosis, internalization, and subsequent intracellular degradation of growth factor receptors. For the Type I cytokine receptors to which the G-CSFR belongs, endocytosis and degradation have been best characterized for the growth hormone receptor (GH-R), interleukin-2 receptor (IL-2R), and the erythropoietin receptor (EpoR) [14]–[19]. These receptors all undergo ligand-induced ubiquitination. Enzymatic attachment of ubiquitin, a 76 amino acid polypeptide, to lysine residues present in these receptors was shown to provide a recognition tag for delivery of the receptors to either the lysosome or proteasome. In the acidic environment of lysosomes, bound ligands can dissociate from their respective receptors, while in the proteasome the ubiquitinated receptors are degraded, processes that both culminate in attenuation of receptor signaling.

We previously examined the role of the ubiquitin/proteasome system in G-CSFR internalization and intracellular sorting [20]. The G-CSFR was shown to be ubiquitinated via ligand-dependent and independent mechanisms and disruption of the ubiquitin machinery inhibited endocytosis of the G-CSFR. Pre-treatment of both transfected cells and primary human neutrophils with proteasome inhibitors inhibited ligand-induced degradation of the activated G-CSFR, indicating the importance of G-CSFR ubiquitination and the proteasome in modulating G-CSFR surface expression.

To further investigate the role of ubiquitination in G-CSFR-mediated cell proliferation, we generated a mutant form of the G-CSFR (K762R/G-CSFR) which abrogates the attachment of ubiquitin molecules to the lysine residue at position 762 of the receptor that is deleted by mutations in the G-CSFR in patients with SCN/AML. In response to G-CSF, mono-/polyubiquitination was impaired in cells transiently expressing the mutant receptor form. Cells stably transfected with the K762R/G-CSFR exhibited increased growth and enhanced sensitivity to G-CSF, and extended cell survival following G-CSF depletion. Activation of both Stat5 and Akt were also prolonged in K762R/G-CSFR transfected cells in response to G-CSF, and their activation remained prolonged even after G-CSF withdrawal. These data indicate that ubiquitination of the C-terminal lysine in the G-CSFR that is detected in patients with SCN/AML is required for normal G-CSFR-mediated cell proliferation and survival.

## Results

### Mono-/polyubiquitination is impaired in cells transfected with the K762R/G-CSFR

**Figure 1:**
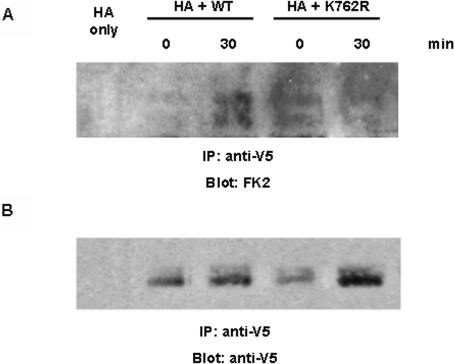
Impaired mono-/polyubiquitination of the K762R/G-CSFR in response to ligand binding. CHO ts20 cells were transiently transfected with HA-tagged ubiquitin alone (HA only), HA-Ubiquitin plus WT G-CSFR (HA+WT), or HA-Ubiquitin plus K762R/G-CSFR (HA+K762R). At 48 hrs after transfection, cells were washed and incubated in serum-free media at 4°C for 4 hrs before being transferred to 37°C and incubated with G-CSF (100 ng/mL) for the indicated times. Cells were then lysed, immunoprecipitated with anti-V5 antibody, and blotted with (A) the FK2 antibody which recognizes both poly- and monoubiquitinated proteins. (B) The blot in (A) was stripped and re-blotted with anti-V5 antibody as a control for receptor protein loading.

We previously reported that the WT G-CSFR undergoes both mono- and polyubiquitination in response to G-CSF, which could be detected using the commercially available FK2 antibody [20]. To investigate whether the K762R/G-CSFR mutation leads to impaired ubiquitination, we transiently co-transfected CHO cells with HA-tagged ubiquitin together with either the wild-type or the mutant G-CSFR construct. As shown in Figure 1A, ligand-induced mono-/polyubiquitination could be detected in cells expressing the WT G-CSFR but not the K762R/G-CSFR mutant. The increased ubiquitination observed in cells expressing the WT G-CSFR but not the K762R mutant could not be attributed to the levels of receptor protein loading as demonstrated by reblotting the same blot with antibody to V5 recognizing the V5 epitope tag fused to each receptor form (Figure 1B). This finding indicates that the lysine at position 762 is critical for ligand-induced ubiquitination of the G-CSFR.

### BaF3 cells expressing the K762R/G-CSFR exhibit increased sensitivity, survival and growth to G-CSF

We previously reported that BaF3 cells transfected with the Δ716 G-CSFR that is detected in a majority of patients with SCN transforming to AML exhibit hyperproliferative responses to G-CSF [10]. Since lysine762 localizes to the region that is deleted in the Δ716 G-CSFR mutant, we were interested in investigating whether cells expressing the K762R/G-CSFR also exhibited ligand-induced hyperproliferative responses to G-CSF.

**Figure 2:**
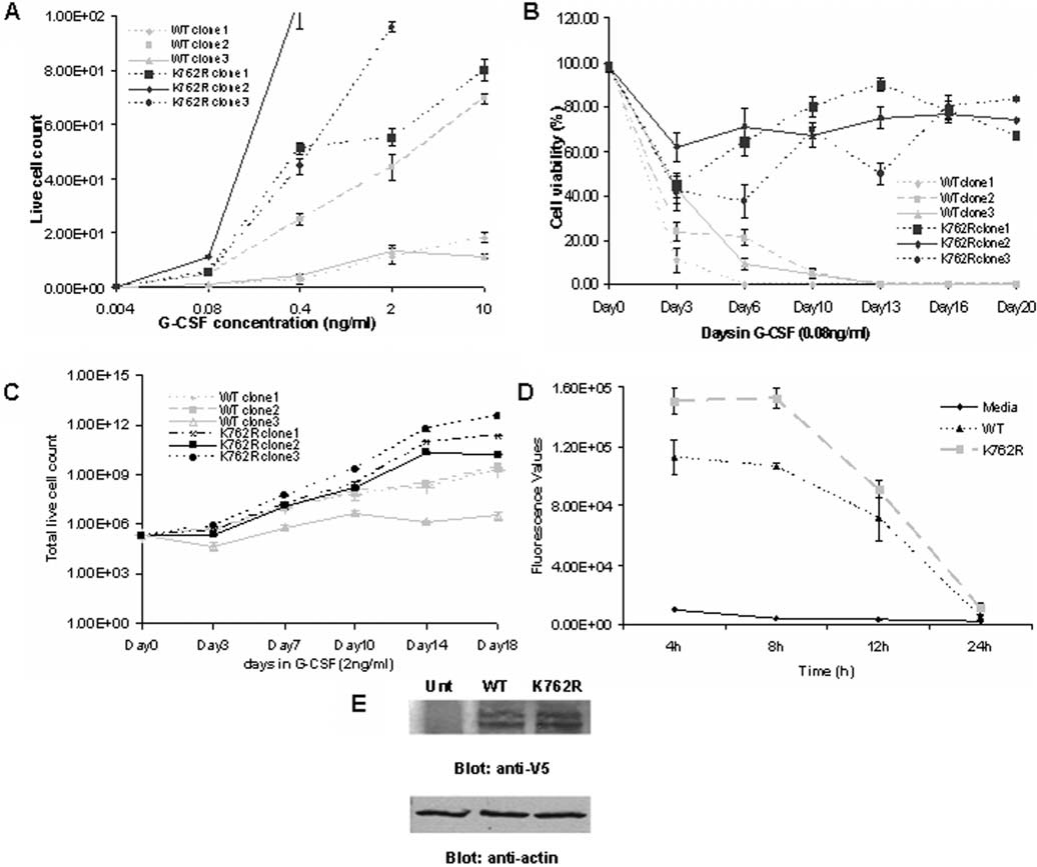
G-CSF hypersensitivity, hyperproliferation, and extended survival of transfected BaF3 cells expressing the K762R/G-CSFR mutant. (A) WT and K762R stable transfectants were grown in varying concentrations of G-CSF for 72 h and cell numbers counted. (B) WT and K762R stable transfectants were grown in 0.08 ng/ml of G-CSF for 20 days and cell viability determined by Trypan Blue staining. (C) Cells were grown in 2 ng/ml of G-CSF for 18 days and cell numbers counted. (D) Pooled transfectants were washed out of IL-3, grown in 10 ng/ml of G-CSF overnight, washed, and the viability of the cells measured over a period of 24 h using the CellTiter-Glo Luminescent Cell Viability Assay. Error bars indicating SEM from three independent experiments are shown. (E) Expression levels of the WT or the K762R G-CSFR were analyzed by Western blotting using anti-V5 antibody (upper panel). Untransfected parental BaF3 cells (Unt) were used as a negative control. The membrane in the upper panel was stripped and re-blotted with anti-actin antibody to confirm equal protein loading (lower panel).

We stably transfected BaF3 cells with either the WT- or the K762R/G-CSFR construct and isolated by limited dilution three independent clones each expressing similar levels of the WT or the K762R/G-CSFR, removed them from IL-3-containing media, washed them, then treated them with varying concentrations of G-CSF for a period of 72 h. As shown in Figure 2A, cells expressing the mutant K762R/G-CSFR grew significantly better than WT G-CSFR-expressing cells at very low concentrations of G-CSF. The shift to the left in the dose-response curve observed with cells expressing the K762R mutant is similar to previously published data from our laboratory with the Δ716 mutant [10], indicating that like the Δ716 mutant, cells expressing the K762R mutant are also hypersensitive to G-CSF. At concentrations of G-CSF as low as 0.08 ng/ml, all WT clones died within 10 days, with some dying as early as 3 days, while all of the K762R clones survived for periods exceeding 20 days (Figure 2B). We next examined the overall growth rate of the BaF3 transfectants at a concentration of G-CSF of 2 ng/ml, a concentration at which both WT and K762R transfectants exhibited long-term growth. For these experiments, the cells were washed out of IL-3, counted, seeded at the same density of 1×10^5^ cells/ml, and grown in 2 ng/ml of G-CSF for a period of 18 days. As shown in Figure 2C, K762R clones proliferated at a higher rate than WT clones.

We next examined the survival capacity of the K762R transfectants compared to WT transfectants following removal from G-CSF-containing media. For these experiments, we used pools of three independent clones of WT transfectants or K762R transfectants and analyzed their survival following incubation in media containing 10 ng/ml of G-CSF and subsequent transfer to cytokine-free media. As shown in Figure 2D, using the ATP-based CellTiter-Glo assay that measures luminescence as a correlation of live cell numbers, K762R-expressing cells exhibited increased survival over WT cells following G-CSF withdrawal. As a control for the CellTiter-Glo assay, results are also shown for wells devoid of cells and containing media alone. To confirm the observed hypersensitivity, hyperproliferation, and prolonged survival of the K762R transfectants was not due to differences in G-CSFR expression levels in the BaF3 transfectants, Western blot analysis using antibody to the V5 tag that was fused to each receptor form was performed. As shown in Figure 2E, equivalent receptor expression levels were detected in WT and K762R transfectants. As an additional control, we further confirmed equivalent protein loading by stripping the same blots and re-blotting them with antibody to actin (Figure 2E, lower panel).

### Myeloid cells expressing the K762R mutant hyperproliferate in response to G-CSF and display increased growth and survival

**Figure 3:**
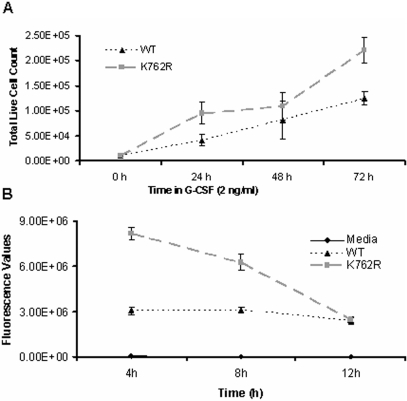
Enhanced proliferation and viability of 32D cells expressing the K762R/G-CSFR mutant. 32Dcl3 cells were stably transfected with the WT G-CSFR or the K762R/G-CSFR. (A) Pooled transfectants were grown in 2 ng/ml of G-CSF and cell numbers determined over a period of 72 h. (B) Pooled transfectants were washed out of IL-3, grown in 10 ng/ml of G-CSF overnight, washed, and the viability of the cells measured using the CellTiter-Glo Luminescent Cell Viability Assay over a period of 12 h. Error bars indicating the SEM from three independent experiments are shown.

To confirm that our observations in the BaF3 lymphoid cell line were physiologically relevant, we next examined the growth and survival of 32D myeloid cells transfected with the K762R mutant. As shown in Figure 3A, 32D cells expressing the K762R/G-CSFR mutant also hyperproliferated in response to G-CSF compared to WT G-CSFR transfectants. Like BaF3 cells transfected with the K762R/G-CSFR mutant, 32D cells expressing the K762R mutant showed enhanced survival following G-CSF withdrawal (Figure 3B).

### Activation of Stat5 but not Stat3 is prolonged in cells expressing the K762R/G-CSFR mutant

**Figure 4:**
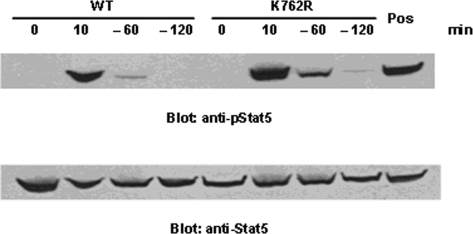
Prolonged G-CSF-induced Stat5 activation in K762R/G-CSFR transfectants. BaF3 cells stably transfected with either the WT G-CSFR or K762R/G-CSFR were washed and incubated in cytokine-free media (RPMI/0.1%BSA) at 37°C for 4 hrs. Cells were then treated with G-CSF (100 ng/ml) at 37°C for 10 min, washed, incubated in media depleted of growth factors for the indicated times, and lysed. Proteins from whole cell lysates were separated on SDS-PAGE and transferred onto nitrocellulose membranes, which were blotted with anti-phospho-Stat5 antibody (upper panel). The blot was stripped and re-blotted with anti-Stat5 antibody to confirm equal protein loading (lower panel). Cells stimulated with activated orthovanadate at room temperature for 20 min are shown as a positive control.

**Figure 5:**
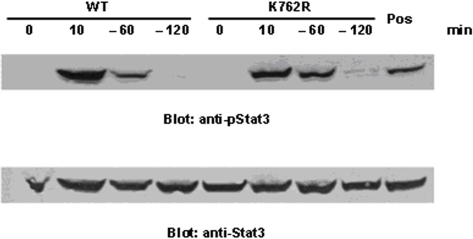
G-CSF-induced Stat3 activation is similar in WT and K762R transfectants. BaF3 cells stably transfected with either the WT G-CSFR or K762R/G-CSFR were washed and incubated in cytokine-free media (RPMI/0.1%BSA) at 37°C for 4 hrs. Cells were then treated with G-CSF (100 ng/ml) at 37°C for 10 min, washed, and incubated again in cytokine-free media for the indicated times, then lysed. Proteins from whole cell lysates were separated on SDS-PAGE and transferred onto nitrocellulose membranes, which were blotted with anti-phospho-Stat3 antibody (upper panel). The blot was stripped and re-blotted with anti-Stat3 antibody to confirm equal protein loading (lower panel). Cells stimulated with activated orthovanadate at room temperature for 20 min are shown as a positive control.

To determine the mechanisms underlying the aberrant response of cells expressing the K762R/G-CSFR mutant to G-CSF, we next examined the activation of Stat5 and Stat3 in these cells, pathways known to be activated by G-CSF. For these experiments, cells were washed out of IL-3-containing media, treated with G-CSF for 10 min, washed, then transferred to cytokine-free media for up to 2 h. Whole cell lysates from WT- and K762R/G-CSFR-transfectants were then analyzed for evidence of phosphorylation of Stat5 and Stat3 by immunoblot analysis with antibodies to phosphor-Stat5 and phosphor-Stat3. As shown in Figure 4, 10 min after G-CSF stimulation, strong phosphorylation of Stat5 was observed in both WT and K762/G-CSFR transfectants. Notably, strong phosphorylation of Stat5 could still be detected in the K762R transfectants compared to WT transfectants at 1 h after G-CSF wash-out, which could still be detected at 2 h. In contrast, in WT cells, Stat5 phosphorylation rapidly diminished after cytokine withdrawal, and could not be detected at 2 h following G-CSF washout. This difference was not due to unequal protein loading as demonstrated by stripping and immunoblotting the same blots with antibody to Stat5 (Figure 4, lower panel). We also examined Stat3 signaling in WT and K762R transfectants following G-CSF stimulation then cytokine wash-out. In contrast to Stat5, no significant differences were detected in WT- and K762R/G-CSFR-transfected cells (Figure 5).

### G-CSF-induced activation of Akt is markedly prolonged in K762R/G-CSFR transfectants

**Figure 6:**
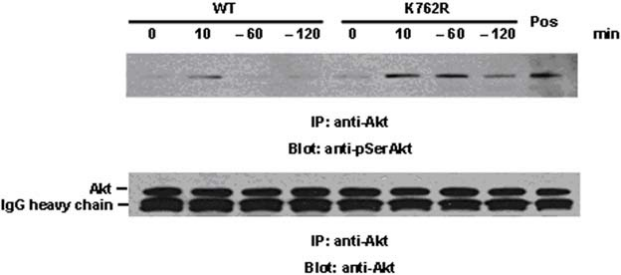
Prolonged Akt activation in response to G-CSF in BaF3 cells transfected with the K762R/G-CSFR. BaF3 cells stably transfected with either the WT G-CSFR or K762R/G-CSFR were washed and incubated in cytokine-free media (RPMI/0.1%BSA) at 37°C for 4 hrs. Cells were then treated with G-CSF (100 ng/ml) at 37°C for 10 min, washed, incubated in cytokine-free media again for the indicated times, and lysed. Whole cell lysates were immunoprecipitated with anti-Akt antibody, and blotted with anti-phospho-Ser-Akt antibody (upper panel). The blot was stripped and re-blotted with anti-Akt antibody to confirm equal protein loading (lower panel). Cells stimulated with activated orthovanadate at room temperature for 20 min are shown as a positive control.

We next investigated activation of the Akt signaling pathway in response to G-CSF, which we have previously shown to be important in G-CSF-mediated cell survival and proliferation. Cells were stimulated with G-CSF for 10 min, lysed, and immunoprecipitated with Akt antibody, then blotted with an antibody recognizing the phospho-serine Akt species. As shown in Figure 6, phosphorylation of Akt on serine residues is readily detected at 10 min following G-CSF stimulation in both WT- and K762R/G-CSFR transfectants. Notably, phosphorylation of Akt on serine residues was markedly prolonged in K762R mutants compared to WT transfectants (Figure 6). Detection of pSerAkt in WT cells was transient and undetectable by 60 min in WT cells, whereas pSerAkt could still be strongly detected in K762R transfectants at both 60 min and 2 h after G-CSF withdrawal.

## Discussion

Receptor desensitization rapidly follows growth factor receptor activation and serves as a mechanism for controlling the amplitude and duration of intracellular signaling. Disruption of the delicate balance between growth factor receptor activation and inactivation has been shown to be associated with severe human pathologies [20]. In the case of the G-CSFR, mutations have been identified in patients with SCN transforming to AML that lead to prolonged receptor activation in response to G-CSF stimulation [9], [21], [22]. These mutations produce C-terminal truncated G-CSFR forms. The Δ716 truncated form of the G-CSFR deletes the distal-most 98 carboxy-terminal amino acids of the G-CSFR and is the most frequent mutation detected in patients with SCN/AML [21]–[25]. Our laboratory and others have previously shown that the mutation in the Δ716 G-CSFR deletes a critical domain that mediates receptor internalization and degradation [9], [26]–[28]. Cells expressing the truncated G-CSFR form exhibit increased receptor expression at the membrane surface, which correlates with sustained intracellular activation and enhanced growth and survival responses to G-CSF [10], [29]–[31].

For many receptors, ligand-induced ubiquitination has been shown to be a crucial initial event in attenuation of receptor signaling [32], [33]. Although the cytoplasmic domain of the G-CSFR lacks a classical ubiquitin-dependent endocytosis motif, it has five lysine residues at positions 632, 672, 680, 681, and 762 that could serve as potential attachment sites for ubiquitin. The lysine residue at position 762 is the sole cytoplasmic lysine that localizes to the region that is deleted in the Δ716 G-CSFR mutant. Recent studies by our laboratory have suggested that defective ligand-induced ubiquitination of the Δ716 G-CSFR underlies its aberrant internalization [20]. We were therefore interested in determining whether site-specific disruption of ubiquitination at lysine762 of the G-CSFR would induce a similar phenotype as the Δ716 G-CSFR truncation mutant.

In the current study, we replaced the lysine residue at position 762 of the G-CSFR with an arginine residue, and examined the effects of this mutation on G-CSF-mediated cellular responses and signaling. CHO cells expressing the K762R/G-CSFR exhibited markedly reduced mono- and polyubiquitination of the G-CSFR in response to G-CSF compared to cells expressing the WT receptor, indicating that lysine 762 is critical for ligand-induced G-CSFR ubiquitination. BaF3 cells stably transfected with the mutant K762R/G-CSFR were found to by hypersensitive to G-CSF and could survive and proliferate in G-CSF concentrations as low as 0.08 ng/ml, a concentration that was insufficient to support the growth and survival of cells expressing the WT receptor. Furthermore, at G-CSF concentrations high enough to support the survival of cells expressing the WT G-CSFR, cells expressing the K762R mutant G-CSFR hyperproliferated in response to G-CSF, which was reproducible in three independent experiments using three different K762R clones. Additionally, following withdrawal of the BaF3 transfectants from G-CSF-containing media, cells expressing the K762R/G-CSFR mutant exhibited prolonged survival over WT G-CSFR-expressing cells. These results suggest that ubiquitination of the G-CSFR at lysine 762 is required for the normal G-CSFR-mediated cell growth and survival.

To confirm the physiologic relevance of our findings in BaF3 cells, we next examined the G-CSF-responsiveness of myeloid cells expressing the K762R mutant. For these experiments, we used the 32Dcl3 cell line. 32Dcl3 cells stably expressing the K762R/G-CSFR mutant also hyperproliferated in response to G-CSF and exhibited enhanced survival following cytokine depletion. These findings are consistent with our previously reported observations with cells expressing the Δ716-G-CSFR [10], and suggest that defective ubiquitination contributes to the aberrant cellular response of cells from patients with SCN/AML to G-CSF.

Previous studies have demonstrated that both Stat3 and Stat5 are rapidly activated and phosphorylated in response to G-CSF [34]. We were therefore interested in determining whether mutation of lysine 762 which disrupts ligand-induced ubiquitination of the G-CSFR had any effect on Stat3 and Stat5 activation. Notably, cells expressing the K762R/G-CSFR exhibited increased activation of Stat5 in response to G-CSF compared to WT cells, and Stat5 activation remained prolonged in K762R-expressing cells even following G-CSF withdrawal. In contrast, there was no evidence that activation of Stat3 was altered in K762/G-CSFR-expressing cells. These findings are consistent with recently published data from Link's group in which expression of a truncated mutant murine G-CSFR form (d715F) in mice that is equivalent to the Δ716 human G-CSFR in patients with SCN/AML was found to confer a strong clonal advantage that was dependent on Stat5, but not Stat3 [35]. Importantly, Stat5 has been implicated in leukemogenesis, and expression of constitutively active Stat5 in hematopoietic cells has been shown to induce a rapidly fatal myeloproliferative disease in mice [36]–[38].

A recent study by Irandoust et al in which all five lysine residues in the G-CSFR were mutated to arginine was reported to result in hyperproliferative responses to G-CSF [34]. These authors also reported that cells expressing the lysine-deficient G-CSFR exhibited prolonged activation of not only Stat5 but Stat3 as well. The reasons for the differences in their results and those reported here are not clear. However, collectively the results suggest that the proximal four lysine residues in the G-CSFR may play roles in Stat3 activation while lysine 762 is critical for Stat5 signaling, implicating distinct roles for individual cytoplasmic lysine residues in the G-CSFR.

Activation of the Akt signaling pathway has been reported to protect a variety of cell types from apoptosis [10]. G-CSF has been shown to induce rapid phosphorylation of Akt at serine 473 in both WT- and Δ716 G-CSFR-expressing cells. Moreover, we have previously reported that the enzymatic activity of Akt in response to G-CSF is prolonged in cells expressing the Δ716 G-CSFR [10]. Our current results also demonstrate enhanced and prolonged activation of Akt in K762R-expressing cells in response to G-CSF compared to WT cells, suggesting that ubiquitination is also involved in regulation of the Akt signaling pathway in response to G-CSF stimulation.

Collectively, our data suggest a role for ubiquitination in G-CSF-mediated cell survival and proliferation. Lysine 762 in the cytoplasmic tail of the G-CSFR appears to be critical in regulating the amplitude and duration of Stat5 and Akt activation. Future studies to identify the molecules that interact with the ubiquitinated G-CSFR and the E3 ligase that is recruited to the phosphorylated G-CSFR should provide additional insights into the mechanisms that downregulate G-CSFR signaling, which are disrupted in patients with SCN/AML.

## Materials and Methods

### Reagents and cell culture

G-CSF-responsive murine 32Dcl3 cells were kindly provided by Dr. Giovanni Rovera (The Wistar Institute, Philadelphia, PA, USA) and were maintained in RPMI-1640 medium supplemented with 10% fetal bovine serum (FBS) and 10% WEHI 3B conditioned medium as a source of IL-3. BaF3 cells were maintained in RPMI 1640 medium supplemented with 2 mM glutamine, 10% FBS, and 10% WEHI-3B conditioned media as a source of interleukin 3 (IL-3). Chinese hamster ovary (CHO) cells were grown in αMEM (αMinimum Essential Media) supplemented with 4.5 g/L glucose and 10% FBS. Penicillin and streptomycin (100 U/mL each) were added to all culture media. Recombinant human G-CSF was a generous gift from Amgen (Thousand Oaks, CA). Media and cell culture reagents were purchased from GIBCO/Invitrogen (Carlsbad, CA).

### Plasmid construction and transfection

The wild type G-CSFR (WT G-CSFR) cDNA was cloned into the pcDNA3.1D-TOPO mammalian expression vector (Invitrogen) in frame with a V5 epitope tag, as described previously [39]. QuikChange® II Site-Directed Mutagenesis Kit was purchased from Stratagene (La jolla, CA), and the K762R/G-CSFR mutant was generated according to the protocol provided with the kit. Briefly, using the WT G-CSFR plasmid as a template, PCR reactions were performed with an initial 30-second denaturation at 95°C, followed by 14 cycles of denaturation at 95°C for 30 seconds, annealing at 55°C for 1 minute, and elongation at 68°C for 8 minutes, and a final digestion by DpnI for 1 hour at 37°C. The forward primer was 5′-CCCCAGCCCCAGGTCCTATGAGAACCTC-3′, and the reverse primer was 5′-GAGGTTCTCATAGGACCTGGGGCTGGGG-3′. The K762R/G-CSFR was also epitope-tagged with V5. The fidelity of the entire open reading frame of each expression vector was confirmed by automated DNA sequencing. The hemagluttinin (HA)-tagged ubiquitin cDNA was generously provided by Dr. Dirk Bohmann (University of Rochester, Rochester, NY).

BaF3 cells were stably transfected with the various G-CSFR forms using conditions that have previously been described [40]. Single clones were isolated by limiting dilution and screened by immunoblotting. A pool of three single clones with similar expression levels was used in the experiments. Stable transfection of 32Dcl3 cells was described previously [41]. Transient transfection of CHO cells was performed in 60 mm dishes using Effectene reagent (Qiagen, Valencia, CA) according to the manufacturer's instructions.

### Cell viability and proliferation

Stably transfected BaF3 and 32Dcl3 cells (WT and K762R mixed-pools) were removed from IL-3-containing media, washed, and then grown in the presence of 10 ng/ml of G-CSF at 37°C overnight. Cells were then washed and resuspended in cytokine-free media and incubated at 37°C for 0 h, 4 h, 8 h, 12 h, and 24 h. At each time point, 5×10^3^ cells were removed and analyzed using the CellTiter-Glo Cell Viability Luminescence Assay protocol (Promega, Madison, WI) utilizing the Berthold Technologies Centro XS^3^ LB 960 plate luminometer and MicroWin 2000 software. Cells were also counted at each time point and cell viability was determined by Trypan Blue staining. For proliferation experiments, cells were grown in different concentrations of G-CSF for 72 h to determine their dose response to G-CSF. Aliquots of cells were subsequently grown in 2 ng/ml of G-CSF for either 72 h (32Dcl3 cells) or 18 days (BaF3 cells) and cell numbers counted at each time point.

### Antibodies, immunoprecipitations and immunoblot analysis

Antibody to V5 was purchased from Invitrogen (Carlsbad, CA). Anti-poly/monoubiquitin antibody raised against the FK2 clone was purchased from Affiniti Research (Exeter, UK). Antibodies to phospho-Stat3 (pStat3), phospho-Stat5 (pStat5), and phospho-serine-Akt (pSerAkt) were purchased from Cell Signal (Danvers, MA). Antibodies to Stat3, Stat5, Akt, and actin were from Santa Cruz (Santa Cruz, CA).

Cells (2×10^7^/mL) were lysed in ice-cold lysis buffer (1.5% Triton X-100, 0.1% Sodium Dodecyl Sulfate (SDS), 0.1 mM sodium deoxycholate, 500 mM NaCl, 5 mM EDTA, 10 mM N-ethylmaleimide, 25 mM HEPES, pH 7.8) containing a cocktail of protease inhibitors (Roche, Indianapolis, IN), incubated on ice for 20 min, and centrifuged at 4°C at 10,000 g. The supernatants were collected from each sample and protein concentrations determined using the BCA reagent (Pierce, Rockford, IL). A total of 400 mg of protein from each sample in equivalent final volumes was mixed 1∶1 with IP buffer (10% glycerol, 100 mM KCl, 5 mM MgCl_2_, 50 mM Tris, pH 8), and incubated with 1–2 µg antibodies and 30 µL of protein G-agarose (Invitrogen) overnight at 4°C. Immunoprecipitates were collected by centrifugation at 2,000 g at 4°C, washed 3× with a 1∶1 mix of lysis and IP buffers, and resuspended in LDS sample buffer (Invitrogen). The samples were heated to 70°C for 10 min, resolved on 4–12% Bis-Tris acrylamide gels using a MOPS running buffer (Invitrogen), and transferred to nitrocellulose. For analysis of whole cell lysates, 20–80 µg of protein from each sample was resolved by SDS-PAGE and subjected to immunoblot analysis. Immunoreactive bands were visualized using the enhanced chemiluminescence (ECL) reagent (Amersham, Piscataway, NJ). For densitometric analysis, ImageJ software made available through the National Institutes of Health was used.

For analysis of G-CSFR ubiquitination in cells transiently co-expressing HA-tagged ubiquitin and either the WT G-GSFR or the K762R/G-CSFR, CHO cells were transferred 48 h after cotransfections to serum-free MEMα media (containing 20 mM HEPES, penicillin/streptomycin, 4.5 gm/L glucose, and 1% BSA) for 4 h at 4°C, and incubated with 100 ng/mL G-CSF at 37°C for varying times. For signaling studies, cells were washed and incubated in serum-free media (RPMI/0.1%BSA) devoid of all cytokines and incubated at 37°C for 4 hours. Cells were then treated with G-CSF (100 ng/ml) at 37°C for 10 min, washed, incubated in serum and cytokine-free media for varying times, and lysed. Aliquots of cells were stimulated with activated orthovanadate (5 µl/100 µl cell suspension) at room temperature for 20 min as a positive control. Cell lysis, immunoprecipitations and immunoblotting were performed as described above.
